# Solitary Sexual Desire: Its Relation to Subjective Orgasm Experience and Sexual Arousal in the Masturbation Context within a Spanish Population

**DOI:** 10.3390/healthcare11060805

**Published:** 2023-03-09

**Authors:** Oscar Cervilla, Eva Jiménez-Antón, Ana Álvarez-Muelas, Pablo Mangas, Reina Granados, Juan Carlos Sierra

**Affiliations:** 1 Mind, Brain, and Behavior Research Center (CIMCYC), University of Granada, 18011 Granada, Spain; ocervilla@ugr.es (O.C.); evajimeneza@correo.ugr.es (E.J.-A.); alvarezm@ugr.es (A.Á.-M.); pablomangas@ugr.es (P.M.); 2 Department of Nursey, Health Sciences Faculty, University of Granada, 18071 Granada, Spain; reina@ugr.es

**Keywords:** solitary sexual desire, sexual arousal, genital response, subjective orgasm experience, solitary masturbation

## Abstract

The tridimensional sexual desire proposal (i.e., dyadic to partner, dyadic to attractive other and solitary) has been empirically supported. However, solitary sexual desire and its relationship to other dimensions of sexual functioning has received less attention. Hence, we examined the capacity of solitary sexual desire to explain the subjective orgasm experience (Study 1) and sexual arousal (Study 2) in the context of solitary masturbation. Study 1, composed of 2406 heterosexual adults (M age = 39.72, SD = 11.81), assessed for solitary sexual desire, dyadic sexual desire, and the intensity of the subjective orgasm experience obtained through solitary masturbation, along with other associated parameters. Study 2, consisting of 41 heterosexual young people (M age = 22.49, SD = 3.17), evaluated the genital response (penile circumference/vaginal pulse amplitude) and subjective arousal to sexually explicit films related to solitary masturbation. In both men and women, solitary sexual desire accounted for a significant percentage of the subjective orgasm experience obtained through solitary masturbation. In addition, in women, the propensity for sexual arousal was explained by solitary sexual desire. It is concluded that solitary sexual desire -as opposed to dyadic- is important to explain sexual arousal and orgasm in the solitary masturbation context. These results highlight the importance of addressing sexual desire in the solitary context, given its implications with other dimensions of sexual functioning.

## 1. Introduction

Sexual desire refers to the interest that someone shows in sexual activity either alone or as a couple, including having sexual thoughts [1,2]. Spector et al. [2] proposed two types of sexual desire: dyadic and solitary. Dyadic sexual desire refers to showing an interest in participating in sexual activity with someone else and could involve the desire to be intimate with or share this with that person. Solitary sexual desire refers to showing an interest in participating in sexual activity with oneself (e.g., masturbation), which could involve the desire to abstain from intimacy or sharing with others. Later, Moyano et al. [3] formulated two types of dyadic sexual desire: one refers to partner-focused dyadic sexual desire, while the other refers to dyadic sexual desire for an attractive (different) person. This tridimensional sexual desire proposal (i.e., dyadic to partner, dyadic to attractive other and solitary) has been empirically supported because all three types have been differently associated with similar constructs, such as genital and subjective sexual arousal [4], subjective orgasm experience during sexual relationships [5] or sexual satisfaction [3]. Apart from Spain, this model has also been supported in different countries like Portugal [6] and Colombia [7] and in populations other than heterosexuals [8].

Sexual arousal refers to the emotional-motivational state that someone experiences and which brings about physiological, cognitive-affective and behavioral changes in the sexual setting [4,9]. It can be studied from the subjective perspective, that is, by referring to the cognitive and affective responses that it causes and based on the triggered genital response or physiological changes [10]. Sierra et al. [4] analyzed the role of sexual desire in objective and subjective sexual arousal experimented in a laboratory using films in which a heterosexual couple is involved in sexual relationships. The results showed that the partner-focused dyadic sexual desire explained a significant percentage of men’s and women’s genital responses. Additionally, the desire for an attractive person was a significant predictor of men’s subjective sexual arousal. However, solitary sexual desire was not associated with objective or subjective sexual arousal. The Dual Control Model proposes that sexual arousal is the result of striking a balance between the inhibitory and excitatory mechanisms of the central nervous system [11], and both mechanisms are relatively independent of one another. This propensity to sexual excitation/sexual inhibition is essential for controlling situations of sexual risk (where high sexual excitation would predominate over low sexual inhibition) and sexual functioning problems (involving greater inhibitory system activation vs. excitatory activation) [12]. Therefore, with this model, sexual arousal is conceived as a state and a trait [13]. The sexual arousal trait (i.e., sexual excitation), unlike the state, is easily evaluated with validated self-report scales [14,15,16], which has facilitated the further study of the relationship with other variables, such as sexual desire [3,7].

Arcos-Romero et al. [1] examined the relationship between the three sexual desire dimensions and subjective orgasm experience in the heterosexual relationship context. They found that subjective orgasm experience was significantly associated with partner-focused dyadic sexual desire but not with either dyadic sexual desire for an attractive person or solitary sexual desire. Arcos-Romero and Sierra [5] reported that partner-focused dyadic sexual desire and sexual satisfaction are significant predictors of subjective orgasm experience.

The sexual desire dimensions are also related to sexual satisfaction. Thus, partner-focused dyadic sexual desire is positively related to sexual satisfaction. Additionally, dyadic sexual desire for an attractive person and solitary sexual desire are negatively related in men (i.e., lower sexual satisfaction would be associated with stronger dyadic desire outside the relationship or with more solitary sexual activity) or are not related at all for women [3].

It is known that a subjective orgasm experience may vary according to the context in which it takes place [17]. Indeed, it has been shown that women who have difficulties with having orgasms in the sexual relationship context are capable of experiencing an orgasm by means of masturbation [18]. To date, and except for the positive correlations reported by Cervilla et al. [19], the existing relationship between solitary sexual desire and the intensity with which orgasms are subjectively experienced has not yet been studied in detail on all its dimensions in the masturbation context. Nor are there any studies that have related solitary sexual desire to objective sexual arousal (genital response) and subjective experience to visual masturbation-related stimuli. 

Subjective orgasm experience in both the sexual relationships and masturbation contexts has been described using four dimensions [19,20]: (1) Affective; associated with the emotions experienced while having an orgasm (e.g., pleasurable); (2) Sensory; related to the perception of physiological orgasm episodes (e.g., pulsating); (3) Intimacy, or the intimate dimension of orgasm experience (e.g., tender); (4) Rewards; associated with the consequences or effects of an orgasm (e.g., soothing).

Previous studies have highlighted the importance of considering covariates that could play a significant role in the subjective orgasm experience, such as age, education level, and having a partner or not [5,17,21,22], as well as variables more specific to the context of masturbation, such as age at first masturbation, frequency of masturbation, and religious frequency [19,23,24,25]. The results found in the previous literature require us to further explore the role of solitary sexual desire and its relevance to other dimensions of sexual functioning. The present descriptive ex-post-facto cross-sectional study [26] was designed in this context and included two independent projects:

(1) Study 1 was designed to determine in men and women the capacity that solitary sexual desire has to explain the different dimensions of subjective orgasm experience in the solitary masturbation context (Objective 1). It is hypothesized (H1) that solitary sexual desire, compared to dyadic sexual desire for an attractive person, would be associated to a greater extent with the intensity of subjective orgasm experience in the masturbation context [1,5].
(2) Study 2 was to determine in men and women the capacity that solitary sexual desire has to explain sexual arousal: objective and subjective sexual arousal experienced with sexual masturbation-related stimuli and the propensity to feel sexually excited/inhibited (Objective 2). To do so, it is hypothesized (H2) that solitary sexual desire, compared to dyadic sexual desire for an attractive person, would be associated with a greater intensity of objective sexual arousal (genital response) and subjective sexual arousal (general sexual arousal and genital sensations) with visual stimuli related to masturbation behavior [4,19]. Moreover, both these types of sexual desire would be associated positively with the propensity to sexual excitation and negatively with the propensity to sexual inhibition (H3) [3,7].

Study 1.

## 2. Materials and Methods

### 2.1. Participants

By means of an incidental quota sampling (sex and age), 2406 Spanish adults (1085 men and 1321 women) between the ages of 18 and 83 years participated. Inclusion criteria were: (a) heterosexual orientation and (b) having solitary masturbation experiences. 

### 2.2. Instruments

The Socio-Demographic and Sexual History Questionnaire. It collects data about sex, age, education level, nationality, partner relationship, sexual orientation, masturbation experience, age when the first masturbation experience occurred, masturbation frequency, and religious practice.

The Spanish version of the Negative Attitudes Toward Masturbation Inventory (NATMI) [27] by Cervilla et al. [28] consists of ten items to be answered on a five-point Likert-type scale from 1 (not at all true for me) to 5 (extremely true for me). Its internal consistency reliability is 0.95, and it demonstrates adequate construct and discriminant evidence of validity. Higher scores indicate a more negative attitude toward masturbation. In the present study, the ordinal alpha was 0.94. 

The Spanish version of the Sexual Desire Inventory (SDI) [2] by Moyano et al. [3] consists of 13 items distributed in three subscales, whose internal consistency reliability ranges between 0.89 and 0.93: Partner-focused dyadic sexual desire, dyadic sexual desire for an attractive person, and solitary sexual desire. Higher scores indicate stronger sexual desire. Only the last two subscales were considered in this study, whose Cronbach’s alpha coefficients were 0.85 in both cases.

The Spanish version of the Orgasm Rating Scale to the solitary masturbation context (ORS) [29] by Cervilla et al. [30] assesses the subjective orgasm experience in the solitary masturbation context through four dimensions: Affective (feelings experienced), Sensory (physiological sensations), Intimacy (the intimate aspect of the orgasm), and Rewards (consequences or gratifying effects). It consists of 25 items that correspond to adjectives that characterize the most recent orgasm experience through solitary masturbation, using a Likert-type scale from 0 (does not describe it at all) to 5 (describes it perfectly). Regarding its internal consistency reliability, it presents values ranging between 0.71 and 0.95. Discriminant and external validity evidence of its measures have been provided [30]. In this study, the ordinal alpha values were 0.95 for the Sensory dimension, 0.93 for the Affective dimension, 0.72 for the Intimacy dimension, and 0.90 for the Rewards dimension.

### 2.3. Procedure

The battery of instruments was applied online and distributed through virtual platforms (Facebook^®^, Twitter^®^, WhatsApp^®^ and email) using LimeSurvey^®^ software. To avoid automatic responses, participants confirmed their access to the survey by answering a security question consisting of a simple random arithmetic operation. Voluntary participation, the anonymity of all participants, and the confidentiality of their data were guaranteed. Prior to participation, volunteers were asked to read and accept the informed consent form describing the type of study and informing them about data privacy and confidentiality. The study was approved by the Ethics Committee of Human Research of the University of Granada (No. 682/CEIH/2018).

### 2.4. Data Analysis

Using MANCOVA, sex differences in sexual desire and subjective orgasm experience were examined, taking into account as covariates age, education level, having a partner, age of first masturbation, masturbation frequency, negative attitude toward masturbation and prayer frequency. Four multiple linear regression models were performed using the Intro method, separately for men and women, to explain each dimension of orgasm from the two types of sexual desire (solitary and dyadic), taking into account the aforementioned covariates. The R program (version 3.6.3) [31] with the RStudio interface (version 1.2.5042) [32] was used. The missForest package was used for missing data (version 1.4) [33], and the Psych package (version 1.9.12.31) was employed to calculate the ordinal alphas [34]. The other statistical analyses were performed with IBM^®^ SPSS^®^ v.22.

## 3. Results

First, the sociodemographic characteristics of the participants are included in Table 1.

According to the approach proposed in the data analysis section, differences in desire and orgasm were analyzed first. Age (Lambda de Wilks’ = 0.95; *F*(6, 1905) = 17.23, *p* < 0.001; *ƞ*_p_^2^ = 0.05), having a partner (Wilks’ Lambda; = 0.98; *F*(6, 1905) = 8.15, *p* < 0.001; *ƞ*_p_^2^ = 0.03), age of first masturbation (Wilks’ Lambda = 0.99; *F*(6, 1905) = 2.19, *p* < 0.05; *ƞ*_p_^2^ = 0.007), masturbation frequency (Wilks’ Lambda = 0.61; *F*(6, 1905) = 200.21, *p* < 0.001; *ƞ*_p_^2^ = 0.39), and negative attitude toward masturbation (Wilks’ Lambda = 0.92; *F*(6, 1905) = 28.88, *p* < 0.001; *ƞ*_p_^2^ = 0.08) were the significant multivariate covariates. Prayer frequency was not significant (*p* < 0.35). Sex had a main effect on the four dimensions of the subjective orgasm experience and sexual desire (Wilks’ Lambda = 0.90; *F*(6, 1905) = 33.71, *p* < 0.001; *ƞ*_p_^2^ = 0.10). Significant differences were observed in all variables. Women presented higher scores (i.e., higher intensity) than men in all dimensions of the subjective orgasm experience. Men showed higher scores than women in both types of sexual desire. Results are shown in Table 2.

In men, the four linear regression models conducted to explain each of the four dimensions of the subjective orgasm experience were significant (Table 3). University education (β = −0.13), current masturbation frequency (β = −0.12), negative attitudes toward masturbation (β = −0.23), in a negative sense, and solitary sexual desire (β = 0.45), in a positive sense, explained 24% of the variance of the Affective dimension of the subjective orgasmic experience (*F*(10, 843) = 28.75; *p* < 0.001). With respect to the Sensory dimension, the current masturbation frequency (β = −0.17), in a negative sense, and solitary sexual desire (β = 0.43) and dyadic sexual desire for an attractive person (β = 0.08), in a positive sense, explained 15% of the variance (*F*(10, 843) = 16.02; *p* < 0.001). In relation to the Intimacy dimension, the current masturbation frequency (β = −0.17), in a negative sense, with age (β = 0.18) and solitary sexual desire (β = 0.34), in a positive sense, explained 11% of the variance (*F*(10, 843) = 11.82; *p* < 0.001). Finally, negative attitudes toward masturbation (β = −0.10), in a negative sense, and solitary sexual desire (β = 0.27), and dyadic sexual desire for an attractive person (β = 0.07), in a positive sense, explained 9% of the variance in the Rewards dimension of the subjective orgasm experience obtained through solitary masturbation (*F*(10, 843) = 9.68; *p* < 0.001).

In women, the four regression models to explain the dimensions of the subjective orgasm experience were also significant (Table 4). For the Affective dimension, the significant variables were current frequency of masturbation (β = −0.14), negative attitudes toward masturbation (β = −0.10), in a negative sense, and solitary sexual desire (β = 0.45), in a positive sense, explaining 17% of the variance (*F*(10, 1054) = 22.81; *p* < 0.001). For the Sensory dimension, the significant variables were frequency of masturbation (β = −0.08), in a negative sense, and negative attitude toward masturbation (β = 0.09) together with solitary sexual desire (β = 0.36), and dyadic sexual desire for an attractive person (β = 0.15), in a positive sense, which explained 15% of the variance (*F*(10, 1054) = 20.24; *p* < 0.001). On the other hand, age (β = 0.17) and solitary sexual desire (β = 0.29) explained, in a positive sense, 10% of the variance of the Intimacy dimension (*F*(10, 1054) = 12.25; *p* < 0.001). Finally, in the Rewards dimension, only the solitary sexual desire (β = 0.27) significantly influenced positively, explaining 7% of the variance of the subjective orgasm experience induced by solitary masturbation (*F*(10, 1054) = 8.90; *p* < 0.001).

## 4. Discussion

Significant differences between men and women were found in all four dimensions of the subjective orgasm experience, in favor of women, and in solitary and dyadic sexual desire for an attractive person, in favor of men. In both men and women, solitary sexual desire is a significant variable in explaining the four dimensions of the subjective orgasm experience in the context of solitary masturbation. On the other hand, dyadic sexual desire was only significant for the Sensory and Rewards dimensions. These results are discussed together with the findings of Study 2 in the General Discussion.

Study 2.

## 5. Materials and Methods

### 5.1. Participants

Forty-one young people (20 men and 21 women) aged 18 to 30 years were recruited. The mean age was 22.95 years (*SD* = 3.73) in men and 22.05 years (*SD* = 3.73) in women (*t* = 0.91; *p* = 0.37). Inclusion criteria were: (a) being of legal age (≥18); (b) having heterosexual orientation, and (c) having solitary masturbation experience. Exclusion criteria were: (a) having a medical problem and/or psychological disorder; (b) having sexual dysfunction; (c) taking medication that could interfere with sexual functioning; (d) drugs and/or alcohol use; (e) history of sexual abuse. Of the sample, 50% of the men and 57.1% of the women reported that they were in a partnered relationship. 

### 5.2. Instruments

The Socio-Demographic and Sexual History Questionnaire assesses age, sex, nationality, sexual orientation, relationship status, medical/psychological/sexual problems, pharmacological treatments, drug/alcohol use, and sexual victimization history. 

The Spanish version of the Sexual Desire Inventory (SDI) [2] by Moyano et al. [3] was used again. As in Study 1, the subscales of Dyadic sexual desire for an attractive person and Solitary sexual desire were used, whose Cronbach’s alpha coefficients were 0.88 and 0.86, respectively, in this study. 

The Spanish version of the Rating of Sexual Arousal (RSA) [35] was administered. It consists of five items with different Likert-type responses depending on the item (e.g., from 1 = No arousal at all to 7 = Extremely sexually aroused). It assesses self-perceived overall level of sexual arousal, genital sensations, sensations of warmth experienced, non-genital physical sensations and sexual concentration, respectively. Its internal consistency reliability is 0.90. Cronbach’s alpha for this study was 0.93. 

The Spanish version of the Rating of Genital Sensations (RGS) [35] was also given. It is formed by a list of 11 descriptions of genital sensations, from no genital sensation to multiple orgasms. It presents adequate validity evidence [35]. 

The Spanish version of the Sexual Inhibition/Excitation Scales-Short Form [14] by Moyano and Sierra [16] was administered. Its 14 items are distributed in three subscales: Sexual excitation, Sexual inhibition due to the threat of performance failure and Sexual inhibition due to the threat of performance consequences of sexual activity. Its internal consistency reliability coefficients range from 0.60 to 0.72, and its subscales have adequate validity evidence [15]. In this study, the ordinal alpha coefficient ranged from 0.61 to 0.88 for the three subscales.

The Biopac Model MP150 system with 16 channels (Biopac Systems Inc., Goleta, CA, USA), with software AcqKnowledge^®^ 5.0. for data acquisition, together with a penile pletismograph module (Biopac amplifier DA100C and indium/gallium plethysmograph sensors) and a vaginal photopletysmography module (Biopac amplifier PPG100C and vaginal transducer) were utilized. 

Three-minute content-neutral and sexually explicit videos with either a man or a woman masturbating were shown. The sexual videos were previously validated, demonstrating the ability to elicit sexual arousal [36].

### 5.3. Procedure

First, to recruit potential participants, we used university mailings, posters, and posts on social networks. Volunteers interested in participating accessed an online survey to complete the screening instruments in order to recruit the participants who met the inclusion criteria. Eligible participants for the experimental task were contacted and made appointments at the Human Sexuality Laboratory located in the Mind, Brain and Behavior Research Center of the University of Granada. The experimental task consisted of viewing five three-minute videos, two with explicit sexual content (a person of the opposite sex masturbating) and three with neutral content. Simultaneously a psychophysiological recording of their genital response was made, and their subjective sexual arousal was evaluated after each film.

The voluntary participation and anonymity of all participants were guaranteed, as well as the confidentiality of their data. They received no reward for their participation. Before responding, they were asked to read and accept the informed consent form describing the type of study and informing them about data privacy and confidentiality. The study was approved by the Ethics Committee of Human Research of the University of Granada (No. 682/CEIH/2018).

### 5.4. Data Analysis

Considering the sample size and that some variables did not have a normal distribution (Kolmogorov-Smirnov and Shapiro-Wilks normality tests) and equality of variances (Levene’s test), nonparametric statistics were used. First, sex differences were examined, using the Mann-Whitney U test, for all variables related to sexual arousal: ratings of sexual arousal, ratings of genital sensations, propensity for sexual excitation, propensity for sexual inhibition due to the threat of performance failure, and sexual inhibition due to the threat of performance consequences. The genital response was considered in terms of increased sexual arousal in response to sexual films with respect to neutral ones. Using Spearman correlations, the relationship between the two types of sexual desire (i.e., solitary and dyadic for an attractive person) and different variables related to sexual arousal were examined in men and women separately. Based on multiple linear regression models, using the Intro method, we examined the extent to which the two types of sexual desire explain sexual arousal in men and women separately. The same statistical programs were used as in Study 1.

## 6. Results

Regarding comparisons in a sexual arousal by sex, no significant differences were observed (Table 5).

Regarding the correlations between the two types of sexual desire and the different variables related to sexual arousal (Table 6), in men, a significant negative correlation was obtained between the dyadic sexual desire for an attractive person and sexual inhibition due to the threat of performance consequences (*r* = −0.48; *p* = 0.03); solitary sexual desire was not associated with any variable related to sexual arousal. For women, solitary sexual desire correlated significantly with genital responsiveness (*r* = 0.50, *p* = 0.02) and propensity for sexual excitation (*r* = 0.63, *p* = 0.002), whereas the dyadic sexual desire for an attractive person correlated only with a propensity for sexual excitation (*r* = 0.47, *p* = 0.03). 

Finally, six multiple linear regression models—for men and women separately—were performed to explain the different variables related to sexual arousal from the two types of sexual desire. In men, only one model was significant in explaining sexual inhibition due to the threat of performance failure (*F*(2, 17) = 3.88; *p* = 0.04). However, neither solitary nor dyadic sexual desire for an attractive person was a significant predictor of sexual desire. The models to explain the genital response (*F*(2, 17) = 1.35; *p* = 0.29), ratings of sexual arousal (*F*(2, 17) = 2.15; *p* = 0.15), ratings of genital sensations (*F*(2, 17) = 0.26; *p* = 0.77), the propensity to sexual excitation (*F*(2, 17) = 2.76; *p* = 0.09), and sexual inhibition due to the threat of performance failure (*F*(2, 17) = 0.13; *p* = 0.88) were not statistically significant.

The models to explain genital response (*F*(2, 18) = 2.04; *p* = 0.16), ratings of sexual arousal (*F*(2, 18) = 0.72; *p* = 0.50), ratings of genital sensations (*F*(2, 18) = 0.61; *p* = 0.55), sexual inhibition due to fear of sexual performance/execution (*F*(2, 18) = 0.20; *p* = 0.82), and sexual inhibition due to fear of consequences (*F*(2, 18) = 1.28; *p* = 0.30) were not statistically significant.

In women, only the model that explains 37% of the variance of the propensity for sexual excitation (*F*(2, 18) = 6.87; *p* = 0.01) from solitary sexual desire (β = 0.49) was significant. This model is summarized in Table 7. The models to explain genital response (*F*(2, 18) = 2.04; *p* = 0.16), ratings of sexual arousal (*F*(2, 18) = 0.72; *p* = 0.50), ratings of genital sensations (*F*(2, 18) = 0.61; *p* = 0.55), sexual inhibition due to the threat of performance failure (*F*(2, 18) = 0.20; *p* = 0.82), and sexual inhibition due to the threat of performance consequences (*F*(2, 18) = 1.28; *p* = 0.30) were not statistically significant.

## 7. Discussion

Study 2 provides evidence about the relationship between sexual desire and objective and subjective sexual arousal to masturbation behavior. In men, significant correlations are observed only between dyadic sexual desire and propensity for sexual inhibition due to the threat of performance consequences; in women, correlations were found between solitary sexual desire and propensity for sexual excitation and genital response, highlighting the ability of solitary sexual desire to explain the propensity for sexual excitation. These results are discussed along with those of Study 1 in the General Discussion.

## 8. General Discussion

Previous studies have explored the capacity of sexual desire to explain sexual arousal [4] and subjective orgasm experience [1] in the sexual relationships context. In both cases, the importance of dyadic sexual desire, as opposed to solitary sexual desire, is stressed. To date, however, the capacity that solitary sexual desire might have to explain, on the one hand, objective and subjective sexual arousal experiences to masturbation-related sexual stimuli and the propensity to feel sexually excited/inhibited and, on the other hand, the different dimensions of subjective orgasm experience in the solitary masturbation context, has not yet been explored. The results obtained from the two performed studies show that solitary sexual desire, compared to dyadic sexual desire for an attractive person, is associated with women’s sexual arousal and plays a key role in explaining subjective orgasm experience in the masturbation context for both men and women. The discussion will follow the sexual response cycle, that is, sexual arousal (Study 2) and orgasm intensity (Study 1).

### 8.1. Comparison of Sexual Desire, Sexual Arousal and Orgasm Experience by Sex

Firstly, sexual differences were examined in the evaluated variables of Study 1 and 2, with significant differences between men and women in the three sexual functioning components (i.e., solitary and dyadic sexual desires for an attractive person, sexual arousal and subjective orgasm experience). 

The differences observed in sexual desire fall in line with what former studies have reported, such that men have indicated higher levels of sexual desire than women [5,37,38]. However, Moyano et al. [3] only found differences between men and women in dyadic sexual desire for an attractive person; this study highlights the importance of distinguishing the different types of sexual desire. These differences might be explained by the bigger sample size and the older mean age of men and women in Study 1. The fact that men reported higher levels of sexual desire could be due to them being better able to communicate more openly than women [39]. Along these lines, Vallejo-Medina et al. [7] indicated that these differences in favor of men could be due to a cultural effect (i.e., women can feel that society places pressure on them to limit their sexual desire and pleasure). Some studies have described how supporting the Sexual Double Standard (SDS) (i.e., the different evaluation of one same sexual behavior depending on if it is performed by a man or a woman) that favors men over women can negatively affect the experience of this desire [40,41]. The involvement of the SDS in sexual desire self-assessment would fall in line with those works that have found a relationship between this attitude and dimensions of sexual functioning, like sexual arousal [42] or sexual satisfaction [43]. Muehlenhard and McCoy [44] have reported that women who support the man’s favorable SDS to a greater extent are more prone to hide their sexual desire from a man during sexual encounters, even though their level of sexual desire is high at the time. 

The fact that there were no significant sex differences between men and women in self-reported excitation when viewing masturbating people coincides with other laboratory studies in which similar masturbation behaviors to those of the present study [45] or sexual relationships [46,47,48] were shown.

Finally, on the differences in subjective orgasm experience, the results back the findings obtained in previous studies that systematically point out how women describe their subjective orgasm experience in both the sexual relationships [20,49,50] and masturbation [19,24,51] contexts more intensely than men. Evidence comparing orgasms in men and women is limited. On the one hand, differences in the structures and functions involved in orgasm in men and women have been described, suggesting that the subjective experience of orgasm may be different [52]. However, other works have also pointed out that there would be more similarities than differences in this sexual response between both [53,54]. Another possible explanation for these differences is that women would include different sensations in their experience on the whole and would attach importance to both affective-emotional aspects and physical or genital reactions [17,55], whereas men would concentrate to a greater extent on their own genital reactions, such as ejaculation, to evaluate their orgasm experience [51].

### 8.2. The Capacity of Sexual Desire to Explain Sexual Arousal and Orgasm Experience

The capacity that solitary sexual desire could have to explain sexual arousal (i.e., propensity for sexual excitation/sexual inhibition, genital response, and general sexual arousal and self-reported genital sensations) (Objective 2) and subjective orgasm experience in the masturbation context (Objective 1) was examined. Regarding H2 and H3, for men, no association appeared between solitary sexual desire and the different variables related to sexual arousal. This result could be explained because sexual arousal involves multiple components, and specifically, in men, it is related or even overlapped with variables such as sexual desire or motivation [9]. Conversely, in women, a positive association was found between solitary sexual desire and vaginal pulse amplitude and the sexual excitation propensity, which accounted for 37% of the variance for the latter. Although the association between solitary sexual desire and vaginal pulse amplitude was moderate, it was not capable of explaining genital response. This was perhaps because the study included only a few women, and therefore, it could be due, rather than to a theoretical aspect, to a mathematical issue related to the number of women in the sample. 

The observed differences between men and women are the importance of solitary sexual desire, as far as its association with the genital response (H2) and its capacity to explain the propensity for sexual excitation (H3), which can be interpreted by the distinct functionality that this type of sexual desire has in both sexes. In women, solitary sexual desire has been related to seeking immediate rewards (e.g., excitation and sexual pleasure), while it is probably more associated with men as a problem with controlling impulses [56]. Likewise, Cervilla and Sierra [19] observed that in women, unlike in men, solitary sexual desire is capable of explaining orgasm satisfaction in sexual relationships. 

As hypothesized (H1) in relation to Objective 1, in both men and women, solitary sexual desire, compared to dyadic sexual desire for an attractive person, is better capable of explaining the intensity of subjective orgasm experience in the masturbation context. Only dyadic sexual desire was associated positively with the Sensorial dimension in men and women, and also with the Rewards dimension in men, and much less intensely than for solitary sexual desire. These results are consistent with those reported by Prause et al. [57], who stated that those women who reported that clitoris stimulation would contribute more to their orgasm also indicated greater solitary sexual desire. Moyano et al. [3] pointed out relative independence among the three sexual desire dimensions insofar as solitary sexual desire and dyadic sexual desire for an attractive person would be expected to show different patterns when associated with subjective orgasm experience in the masturbation context, which was actually the case. Thus, it would be logical for solitary sexual desire to play a more relevant role than a dyadic sexual desire to explain the intensity of subjective orgasm experience obtained by means of masturbation, along the same lines as dyadic sexual desire does in the sexual relationship context [1,58].

### 8.3. Implication of Covariates: Attitude toward Masturbation, Masturbation Frequency and Age

Albeit less importantly than solitary sexual desire, other variables are significant in regression models of subjective orgasm experience obtained through masturbation. Taking a negative attitude toward masturbation was negatively related to the Affective dimension in men and women, to the Rewards dimension in men, and to the Sensorial dimension in women. Previous studies have provided evidence about an association between taking a negative attitude toward masturbation and orgasm dysfunctions, particularly for women [59,60,61]. 

Moreover, masturbation frequency has been associated negatively with the intensity of the subjective orgasm experience acquired by solitary masturbation (specifically on the Affective and Sensorial dimensions for men and women, and on the Intimacy dimension for men). It has been previously indicated that masturbation frequency positively predicts orgasm pleasure in the masturbation context [62,63]. According to these findings, a positive association can be expected with subjective orgasm experience, which was not the case in the current study. Rowland et al. [63] argued that not only masturbation frequency can influence orgasms, but so can the reasons for practicing it; for example, women who frequently masturbate to obtain sexual pleasure are more prone to more intensely experience orgasm experiences than women who masturbate to combat anxiety. Bearing in mind that relieving stress features among the reasons for masturbating [61,63,64,65], masturbating for this reason could be associated with a less intense orgasm experience. Future studies should deal with the relation between reasons for masturbating and the intensity with which orgasms are subjectively experienced. Moreover, sexual behaviors are not exempt from basic learning processes, such as habituation of repeated stimulation [66], which would mean more frequent masturbation would be associated with lesser intensity of subjective experience. It would be interesting for future studies to evaluate the type of self-stimulation and variations that participants include when masturbating to control this habituation process.

Finally, age has been positively associated in men and women with the Intimacy dimension of subjective orgasm experience. Although age has long since been known to negatively impact sexual functioning in general [67,68,69] and subjective orgasm experience in particular [21,70]. Rowland et al. [63,71] also observed a positive relationship between age and orgasm pleasure during masturbation and argued that the more acquired the masturbation experience is, the easier pleasurable orgasms can be achieved. 

### 8.4. Limitations

We must point out some limitations to our studies. First of all, the samples of both our studies were recruited by non-probability sampling, which limits the generalizability of the results. In the population survey study, the evaluation was with an online format, which would involve participants having to be users of social networks to access the survey. As sampling was limited exclusively to cisgender heterosexual people, future studies should include people with other sexual orientations. Finally, the employed experimental design and analysis type did not allow causal relations to be established.

### 8.5. Practical and Theoretical Implications

These findings have practical and clinical implications concerning sexual health. On the one hand, these results provide evidence of the three-dimensional model of sexual desire, consolidating its usefulness for assessing and conceptualizing sexual desire in its solitary dimension. In addition, it is suggested that sexual desire could play a different role depending on the context in which it is assessed, an aspect to be taken into account in this field of research. These studies also show an advance in the investigation of solitary masturbation, and the role of the sexual functioning components in this specific context.

Masturbation is very useful in the field of sex therapy. These results open the door to further study how sexual desire may be associated with sexual arousal and orgasm. The development of studies in this context is essential to have more ways to design more effective interventions. Future work should address in depth the role of solitary sexual desire in improving sexual functioning.

## 9. Conclusions

Despite these limitations, the results evidence the association between solitary sexual desire, particularly in women, and sexual arousal related to masturbation behavior. Furthermore, this type of sexual desire is stressed in relation to dyadic sexual desire for an attractive person to be able to explain subjective orgasm experience in the solitary masturbation context. With all these findings, we emphasize the importance of solitary sexual desire in the sexual therapy setting to deal with other sexual functioning components, such as deficits in sexual arousal and orgasms. Solitary sexual desire, therefore, is a dimension that can enhance sexual health by promoting self-discovery and sexual pleasure. 

## Figures and Tables

**Table 1:** Sociodemographic characteristics of the participants in Study 1.

Variables	Total *n* = 2406	Men *n* = 1085	Women *n* = 1321
Age M (SD)	39.72 (11.81)	40.77 (12.56)	38.87 (11.11)
Education level n (%)			
Primary Education	105 (5.5)	50 (5.9)	55 (5.2)
Secondary Education	646 (33.7)	300 (35.1)	346 (32.5)
University Degree (ongoing or completed)	1168 (60.8)	504 (59.0)	664 (62.3)
Praying frequency n (%)			
Less than once a month	1344 (70)	594 (69.6)	750 (70.4)
Once a month	199 (10.4)	87 (10.2)	112 (10.5)
A few times a month	22 (1.1)	8 (0.9)	14 (1.3)
Once a week	90 (4.7)	33 (3.9)	57 (5.4)
A few times a week	10 (0.5)	1 (0.1)	9 (0.8)
Once a day	104 (5.4)	47 (5.5)	57 (5.4)
More than once a day	95 (5)	54 (6.3)	41 (3.8)
Less than once a month	55 (2.9)	30 (3.5)	25 (2.3)
Partner relationship n (%)			
Yes	1537 (80.1)	723 (84.7)	814 (76.4)
No	382 (19.9)	131 (15.3)	251 (23.6)
Age of first masturbation experience M (SD)	14.80 (5.24)	12.92 (2.37)	16.31 (6.31)
Negative attitude toward masturbation M (SD)	10.93 (2.42)	11.28 (2.95)	10.64 (1.84)
Current masturbation frequency n (%)			
Less than once a month	59 (3.1)	24 (2.8)	35 (3.3)
Once a month	172 (9)	41 (4.8)	131 (12.3)
A few times a month	53 (2.8)	14 (1.6)	39 (3.7)
Once a week	413 (21.5)	109 (12.8)	304 (28.5)
A few times a week	144 (7.5)	52 (6.1)	92 (8.6)
Once a day	791 (41.2)	399 (46.7)	392 (36.8)
More than once a day	208 (10.8)	158 (18.5)	50 (4.7)
Less than once a month	79 (4.1)	57 (6.7)	22 (2.1)

**Table 2:** Sex differences in the four dimensions of the subjective orgasm experience and in the two types of sexual desire.

Variables M (SD)	Men *n* = 854	Women *n* = 1065	*F*_(1, 1910)_	*p*	*d*
Affective Dimension ORS	24.96 (5.03)	26.87 (4.07)	87.31	<0.001	−0.42
Sensory Dimension ORS	31.36 (16.11)	39.13 (15.69)	118.85	<0.001	−0.49
Intimacy Dimension ORS	7.52 (3.64)	8.22 (3.74)	28.34	<0.001	−0.19
Rewards Dimension ORS	11.24 (3.41)	11.71 (3.50)	13.06	<0.001	−0.14
Solitary sexual desire	20.65 (6.10)	19.28 (6.55)	8.21	0.004	0.22
Dyadic sexual desire for an attractive person	10.21 (3.70)	8.86 (3.93)	25.38	<0.001	0.35

Note. ORS: Orgasm Rating Scale.

**Table 3:** Multiple linear regression models for the four dimensions of the subjective orgasm experience in men.

Predictors	B	SE	β	95% CI	*t*	*p*	*R* ^2^	VIF
Affective Dimension							0.24	
Age	−0.02	0.01	−0.04	−0.04, 0.01	−1.13	0.257		1.29
Secondary Education	−1.03	0.67	−0.10	−2.35, 0.29	−1.53	0.127		4.60
University Education	−1.30	0.66	−0.13	−2.59, −0.01	−1.97	0.049		4.67
Having a partner	−0.31	0.43	−0.02	−1.16, 0.54	−0.72	0.474		1.09
Prayer frequency	0.09	0.07	0.04	−0.05, 0.23	1.23	0.221		1.10
First masturbation experience	0.04	0.06	0.02	−0.09, 0.17	0.58	0.560		1.07
Current frequency of masturbation	−0.58	0.20	−0.12	−0.97, −0.18	−2.87	0.004		1.87
Negative attitudes toward masturbation	−0.39	0.05	−0.23	−0.49, −0.28	−7.07	<0.001		1.16
Solitary sexual desire	0.37	0.04	0.45	0.30, 0.44	10.65	<0.001		2.02
Dyadic sexual desire for an attractive person	−0.02	0.05	−0.02	−0.11, 0.07	−0.52	0.602		1.28
Sensory Dimension							0.15	
Age	0.02	0.05	0.2	−0.07, 0.11	0.48	0.631		1.29
Secondary Education	−0.19	2.29	−0.01	−4.67, 4.30	−0.08	0.936		4.60
University Education	−1.25	2.23	−0.04	−5.64, 3.13	−0.56	0.575		4.67
Having a partner	−0.07	1.47	−0.00	−2.96, 2.82	−0.05	0.962		1.09
Prayer frequency	−0.25	0.25	−0.03	−0.74, 0.24	−0.99	0.321		1.10
First masturbation experience	0.26	0.22	0.04	−0.18, 0.70	1.15	0.252		1.07
Current frequency of masturbation	−2.62	0.68	−0.17	−3.96, −1.28	−3.85	<0.001		1.87
Negative attitudes toward masturbation	−0.10	0.19	−0.02	−0.47, 0.27	−0.54	0.591		1.16
Solitary sexual desire	1.14	0.12	0.43	0.90, 1.37	9.56	<0.001		2.02
Dyadic sexual desire for an attractive person	0.33	0.16	0.08	0.03, 0.64	2.14	0.033		1.28
Intimacy Dimension							0.11	
Age	0.05	0.01	0.18	0.03, 0.07	4.83	<0.001		1.29
Secondary Education	0.43	0.53	0.06	−0.61, 1.47	0.82	0.414		4.60
University Education	0.20	0.52	0.03	−0.81, 1.21	0.39	0.699		4.67
Having a partner	0.15	0.34	0.02	−0.52, 0.82	0.45	0.656		1.09
Prayer frequency	−0.04	0.06	−0.02	−0.15, 0.07	−0.67	0.503		1.10
First masturbation experience	0.00	0.05	0.00	−0.10, 0.10	0.05	0.964		1.07
Current frequency of masturbation	−0.60	0.16	−0.17	−0.91, −0.30	−3.80	<0.001		1.87
Negative attitudes toward masturbation	−0.05	0.04	−0.04	−0.14, 0.03	−1.24	0.216		1.16
Solitary sexual desire	0.20	0.03	0.34	0.15, 0.26	7.33	<0.001		2.02
Dyadic sexual desire for an attractive person	0.02	0.04	0.02	−0.05, 0.09	0.60	0.552		1.28
Rewards Dimension							0.09	
Age	−0.01	0.01	−0.02	−0.03, 0.02	−0.51	0.613		1.29
Secondary Education	0.33	0.50	0.05	−0.66, 1.30	0.65	0.516		4.60
University Education	0.15	0.49	0.02	−0.81, 1.12	0.30	0.763		4.67
Having a partner	−0.19	0.32	−0.02	−0.82, 0.44	−0.59	0.558		1.09
Prayer frequency	−0.05	0.05	−0.03	−0.16, 0.06	−0.95	0.342		1.10
First masturbation experience	0.06	0.05	0.04	−0.04, 0.15	1.19	0.235		1.07
Current frequency of masturbation	−0.18	0.15	−0.05	−0.47, 0.12	−1.18	0.238		1.87
Negative attitudes toward masturbation	−0.12	0.04	−0.10	−0.20, −0.04	−2.82	0.005		1.16
Solitary sexual desire	0.15	0.03	0.27	0.10, 0.20	5.57	<0.001		2.02
Dyadic sexual desire for an attractive person	0.07	0.03	0.07	0.00, 0.13	1.98	0.048		1.28

Note. B: non-standardized beta; SE: standard error; β: standardized beta; 95% IC: confidence interval 95%; VIF: variance inflation factor.

**Table 4:** Multiple linear regression models for the four dimensions of the subjective orgasm experience in women.

Predictors	B	SE	β	95% CI	*t*	*p*	*R* ^2^	VIF
Affective Dimension							0.17	
Age	0.00	0.01	0.01	−0.02, 0.03	0.35	0.727		1.27
Secondary Education	0.04	0.55	0.00	−1.04, 1.11	0.07	0.944		5.08
University Education	0.18	0.54	0.02	−0.89, 1.14	0.33	0.744		5.37
Having a partner	−0.07	0.29	−0.01	−0.63, 0.45	−0.23	0.817		1.14
Prayer frequency	0.00	0.06	0.00	−0.12, 0.12	0.02	0.988		1.08
First masturbation experience	−0.03	0.02	−0.04	−0.06, 0.01	−1.43	0.152		1.08
Current frequency of masturbation	−0.60	0.16	−0.14	−0.92, −0.28	−3.66	<0.001		1.88
Negative attitudes toward masturbation	−0.21	0.07	−0.10	−0.34, −0.08	−3.25	0.001		1.10
Solitary sexual desire	0.28	0.03	0.45	0.29, 0.33	11.04	<0.001		2.09
Dyadic sexual desire for an attractive person	0.04	0.03	0.04	−0.03, 0.10	1.12	0.262		1.27
Sensory Dimension							0.15	
Age	0.01	0.05	0.01	−0.08, 0.10	0.27	0.790		1.27
Secondary Education	−1.13	2.13	−0.03	−5.30, 3.05	−0.53	0.596		5.08
University Education	−1.48	2.12	−0.05	−5.64, 2.67	−0.70	0.484		5.37
Having a partner	1.87	1.11	0.05	−0.31, 4.05	1.68	0.093		1.14
Prayer frequency	−0.10	0.24	−0.01	−0.57, 0.37	−0.41	0.682		1.08
First masturbation experience	−0.01	0.07	−0.01	−0.16, 0.13	−0.16	0.872		1.08
Current frequency of masturbation	−1.39	0.64	−0.08	−2.64, −0.14	−2.18	0.030		1.88
Negative attitudes toward masturbation	0.77	0.25	0.09	0.27, 1.26	3.05	0.002		1.10
Solitary sexual desire	0.87	0.10	0.36	0.68, 1.06	8.88	<0.001		2.09
Dyadic sexual desire for an attractive person	0.56	0.13	0.15	0.35, 0.84	4.69	< 0.001		1.27
Intimacy Dimension							0.10	
Age	0.06	0.01	0.17	0.04, 0.08	5.28	<0.001		1.27
Secondary Education	−0.17	0.53	−0.02	−1.20, 0.86	−0.32	0.749		5.08
University Education	−0.10	0.52	−0.01	−1.12, 0.93	−0.19	0.851		5.37
Having a partner	−0.22	0.27	−0.03	−0.76, 0.32	−0.80	0.422		1.14
Prayer frequency	−0.04	0.06	−0.02	−0.16, 0.08	−0.66	0.512		1.08
First masturbation experience	−0.01	0.02	−0.02	−0.05, 0.03	−0.54	0.593		1.08
Current frequency of masturbation	−0.19	0.16	−0.05	−0.50, 0.12	−1.18	0.237		1.88
Negative attitudes toward masturbation	0.04	0.06	0.02	−0.08, 0.17	0.69	0.492		1.10
Solitary sexual desire	0.16	0.02	0.29	0.12, 0.21	6.75	<0.001		2.09
Dyadic sexual desire for an attractive person	0.05	0.03	0.05	−0.02, 0.11	1.45	0.147		1.27
Rewards Dimension							0.07	
Age	0.02	0.01	0.06	−0.00, 0.39	1.68	0.094		1.27
Secondary Education	−0.19	0.50	−0.03	−1.17, 0.79	−0.39	0.699		5.08
University Education	−0.46	0.50	−0.06	−1.43, 0.51	−0.93	0.351		5.37
Having a partner	0.08	0.26	0.01	−0.43, 0.59	0.29	0.769		1.14
Prayer frequency	0.01	0.06	0.01	−0.10, 0.12	0.25	0.805		1.08
First masturbation experience	0.00	0.02	0.01	−0.03, 0.04	0.19	0.852		1.08
Current frequency of masturbation	−0.14	0.15	−0.04	−0.44, 0.15	−0.97	0.334		1.88
Negative attitudes toward masturbation	−0.03	0.06	−0.01	−0.14, 0.09	−0.47	0.641		1.10
Solitary sexual desire	0.15	0.02	0.27	0.10, 0.19	6.38	<0.001		2.09
Dyadic sexual desire for an attractive person	0.02	0.03	0.03	−0.04, 0.08	0.78	0.434		1.27

Note. B: non-standardized beta; SE: standard error; β: standardized beta; 95% IC: confidence interval 95%; VIF: variance inflation factor.

**Table 5:** Differences by sex in sexual arousal.

Variables Mdn (IQR)	Men *n* = 20	Women *n* = 21	*U* Mann Whitney	*p*	*d*
Rating of sexual arousal	10.25 (4.25)	9.25 (9.25)	197.50	0.744	-
Rating of genital sensations	2.25 (2.38)	2.50 (2.38)	198.50	0.967	-
Sexual excitation	16.5 (4.75)	15.00 (4.00)	207.50	0.948	-
Sexual inhibition due to the threat of performance failure	7.50 (3.75)	8.00 (3.75)	194.00	0.672	-
Sexual inhibition due to the threat of performance consequences	11.00 (4.50)	11.00 (3.75)	204.00	0.874	-

Note. Mdn: Median. IQR: Interquartile range.

**Table 6:** Correlations between sexual desire (solitary and dyadic toward an attractive person) and sexual arousal.

Variables	1	2	3	4	5	6	7	8
1. Solitary sexual desire		0.52 *	0.19	0.21	0.01	0.39	0.24	−0.19
2. Dyadic sexual desire for an attractive person	0.42		0.15	0.27	0.06	0.44	0.03	−0.48 *
3. Genital response	0.50 *	0.07		0.77 **	0.77 **	−0.20	0.22	00.02
4. Rating of sexual arousal	0.26	0.28	0.42		0.47 *	0.08	0.19	−0.37
5. Rating of genital sensations	0.31	0.29	0.59 **	0.87 **		−0.23	0.02	0.04
6. Sexual excitation (SES)	0.63 **	0.47 *	0.49 *	0.42	0.46 *		−0.09	−0.26
7. Sexual inhibition due to the threat of performance failure (SIS1)	−0.00	0.15	−0.18	0.03	0.02	0.22		0.11
8. Sexual inhibition due to the threat of performance consequences (SIS2)	0.23	−0.22	0.13	0.16	0.10	0.13	0.45 *	

Note. Values above the diagonal correspond to men and those below to women. * *p* < 0.05; ** *p* < 0.01.

**Table 7:** Multiple linear regression model to explain the propensity for sexual excitation in women.

Predictors	B	SE	β	95% CI	*t*	*p*	*R* ^2^	VIF
Sexual excitation propensity							0.37	
Solitary sexual desire	0.24	0.09	0.49	0.04, 0.44	2.54	0.02		1.18
Dyadic sexual desire	0.15	0.10	0.29	−0.06, 0.365	1.49	0.15		1.18

## Data Availability

The data presented in this study are available on request from the corresponding author. The data are not publicly available due to privacy.
